# Voxel-wise meta-analysis of structural changes in gray matter of Parkinson’s disease patients with mild cognitive impairment

**DOI:** 10.1590/1414-431X20209275

**Published:** 2020-05-18

**Authors:** B. Qin, M.X. Yang, W. Gao, J.D. Zhang, L.B. Zhao, H.X. Qin, H. Chen

**Affiliations:** 1 Department of Neurology, Affiliated Liuzhou People’s Hospital of Guangxi University of Science and Technology/Liuzhou People’s Hospital, Liuzhou, Guangxi, China; 2 Department of Neurology, Yongchuan Hospital of Chongqing Medical University, Chongqing, China

**Keywords:** Voxel-based morphometry, Gray matter, Meta-analysis, Parkinson’s disease, Mild cognitive impairment

## Abstract

Evidence from previous voxel-based morphometry (VBM) studies indicates that widespread brain regions are involved in Parkinson’s disease with mild cognitive impairment (PD-MCI). However, the spatial localization reported for gray matter (GM) abnormalities is heterogeneous. The aim of the present study was to quantitatively integrate studies on GM abnormalities observed in PD-MCI in order to determine whether a pattern exists. Eligible whole-brain VBM studies were identified by a systematic search of articles in PubMed and EMBASE databases spanning from 1995 to January 1, 2019. A meta-analysis was performed to investigate regional GM abnormalities in PD-MCI. The anisotropic effect size version of seed-based d mapping (AES-SDM) meta-analysis was conducted to explore the GMV differences of PD-MCI compared with PD patients with normal cognitive function (PD-NC). A total of 12 studies comprising 243 PD-MCI patients and 326 PD-NC were included in the meta-analysis. PD-MCI patients showed a robust GM decrease in the left insula and left superior temporal gyrus. Moreover, meta-regression analysis demonstrated that age, PD duration and stage, and Unified Parkinson’s Disease Rating Scale III and Mini-Mental State Examination scores might be partly correlated with the GM abnormalities observed in PD-MCI patients. The convergent findings of this quantitative meta-analysis revealed a characteristic neuroanatomical pattern in PD-MCI. The findings provide some evidence that MCI in PD may result in the breakdown of the insula and temporal gyrus, which may serve as specific regions of interest for further investigations.

## Introduction

Parkinson’s disease (PD) is the most common chronic neurodegenerative disease and may lead to mild cognitive impairment (MCI) and dementia (1). The estimated prevalence of PD with dementia (PDD) ranges from 10 to 30%, and the risk of dementia is almost six times higher in PD patients than in the general population (2,3). PD patients without dementia but with mild cognitive impairment (i.e., PD-MCI) are at increased risk of developing dementia (4–6). As in Alzheimer’s disease, patients with PD-MCI have an increased risk of developing dementia compared with those without the condition (5,6). Thus, cognitive dysfunction is a common and debilitating feature of PD; moreover, MCI is an early manifestation of dementia in PD patients. However, the factors contributing to cognitive dysfunction in PD are not completely understood.

Neuroimaging is useful for investigating brain structural features of PD-MCI patients. Voxel-based morphometry (VBM) is a fully automated, quantitative magnetic resonance imaging (MRI)-associated processing method for detecting subtle morphological and neuropathological changes in the whole brain and is used to quantify changes in gray matter volume (GMV) in neuropsychiatric and neurological disorders such as PD and PDD (7). VBM has also been applied to the investigation of GM atrophy in PD-MCI; however, the findings have been contradictory. For instance, GM loss has been reported in cortical and subcortical regions mainly in the left middle frontal gyrus, precentral gyrus, left superior temporal lobe, and right inferior temporal lobe of PD-MCI patients (8), whereas another study found no differences in GM volume between PD patients with normal cognitive function (PD-NC) and those with PD-MCI (9). Furthermore, meta-analyses comparing PD-MCI and PD-NC patients found GM atrophy in the left superior temporal lobe, left insula, and left superior frontal lobe, which was linked to cognitive impairment (10), as well as reduced GMV in the left insula and left superior frontal lobe in PD-MCI patients (11). However, other investigations failed to detect any GM atrophy related to cognitive impairment (12,13). These reported discrepancies could be due to the limited number of studies included in previous meta-analyses; recently, there have been more studies using VBM to investigate changes in brain structure in PD-MCI patients.

Anisotropic effect size-based signed differential mapping (AES-SDM) is a newly developed meta-analysis tool for neuroimaging data that has been applied to examinations of multiple disorders, including obsessive-compulsive disorder and bipolar disorder, and others (14–16), as well as to VBM studies on GMV in PD and other neurological diseases (17,18). AES-SDM allows exhaustive and accurate meta-analysis by employing standard effect size and variance-based calculations in addition to combining both peak coordinates and statistical parametric maps. It also shows good overlap with pool analysis and has adequate sensitivity and excellent control of false positives (19). In order to establish a consistent and reliable GM map in PD-MCI patients, here we carried out a comprehensive meta-analysis of recent VBM studies using AES-SDM. Our findings provided insights into the neuroanatomical substrates and pathophysiological mechanisms of PD-MCI that could improve diagnosis and treatment.

## Material and Methods

### Literature search

Systematic and comprehensive searches of articles in PubMed and EMBASE databases published between 1995 and January 1, 2019 were performed using the following keywords: “voxel” or “VBM or morphometry or gray matter or grey matter” AND “cognitive impairment or mci or cognitive decline or cognition” AND “Parkinson’s disease or Parkinson OR PD”. A manual search of the reference lists of relevant articles was performed to identify additional potential studies.

### Study selection and data extraction

**Figure 1:**
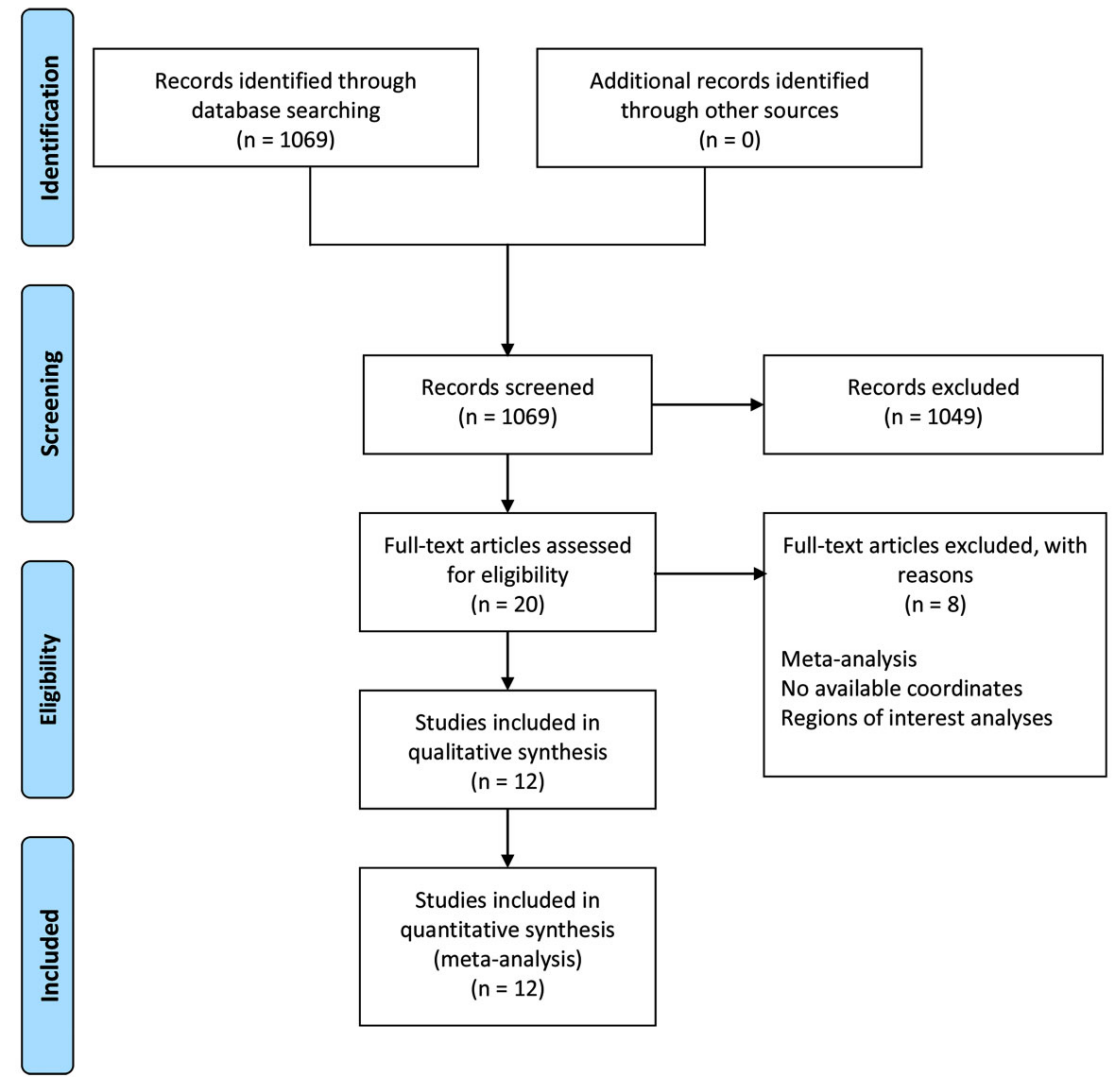
Search strategy used for the inclusion of studies that were considered for the meta-analysis.

Studies were included in the meta-analysis if they met the following criteria: 1) the patient group included PD-MCI or PD-NC patients; 2) VBM studies reported a voxel-wise comparison of GM density or volume between PD-MCI and PD-NC patients; 3) whole-brain results in three-dimensional coordinates (x, y, z) of changes in standard stereotactic space (Talairach or Montreal Neurological Institute) were reported; 4) significance thresholds corrected for multiple comparisons or uncorrected with spatial extent thresholds were used; 5) the sample size in each group was more than 5; and 6) studies were published in English as a peer-reviewed article (not as a letter or abstract). The exclusion criteria were as follows: 1) sufficient data for the meta-analysis could not be obtained even after corresponding with the authors by email; 2) findings were based solely on small volume correction; and 3) region of interest methods were used. Three researchers independently performed the study selection and data extraction and any discrepancies were resolved by consensus through discussion. The study followed the guidelines of preferred reporting items for systematic reviews and meta-analyses (Figure 1).

### Statistical analyses and data preparation

Regional differences in GM changes between PD-MCI and PD-NC patients were analyzed using AES-SDM software (version 4.31, http://www.sdmproject.com), a new version of SDM that has been described in detail elsewhere (19,20). The analytical processes were referred to the AES-SDM tutorial and guidelines. First, coordinates of cluster peaks and effect-sizes (derived from e.g., t-values, or equivalently from P-values or z-scores) were extracted from each data set according to AES-SDM inclusion criteria. An effect size signed map of the differences in GM was separately recreated for each study. Full effect sizes were assigned to peaks (positive for GM increases and negative for decreases), and decreasing effect sizes were assigned to voxels that showed a lower correlation with the peak (20). Second, a mean map was obtained by voxel-wise calculation of the random-effects mean of study maps that were weighted by the sample size and variance of each study. Any potential heterogeneity associated with the main coordinates were also assessed by AES-SDM. Finally, a more stringent significance threshold was set to uncorrected P<0.001 (empirically equivalent to P<0.05, corrected) and a cluster-level threshold of 10 voxels (21). Additional jackknife sensitivity analyses were also carried out by excluding one sample at a time in order to evaluate the robustness of the results across different studies.

Meta-regression analysis was performed to determine the correlation between clinical variables (e.g., age, disease duration, education level, Mini-Mental State Examination [MMSE] score, and Unified Parkinson’s Disease Rating Scale [UPDRS]-III score) and GMV changes that could contribute to the heterogeneity of the results. We used a voxel threshold of P<0.0005 and cluster extent=10 voxels in the meta-regression analyses. Maps showing statistical significance were visualized using MRIcron (https://www.mccauslandcenter.sc.edu/crnl/mricro).

## Results

After an initial assessment of 1069 relevant studies identified with our search strategy, 20 VBM studies of GM changes in PD-MCI patients were identified. Eight of these were excluded for the following reasons: 1) use of a regions of interest analyses; 2) no available coordinates; and 3) published as a meta-analysis. Thus, 12 studies comprising 243 PD-MCI and 326 PD-NC patients were ultimately included in the meta-analysis (8,9,11–13,22–28). Demographic and clinical characteristics for each study that met the inclusion criteria and neuroimaging parameters are outlined in Supplementary Table S1.

### Pooled meta-analysis of regional GM changes

**Figure 2:**
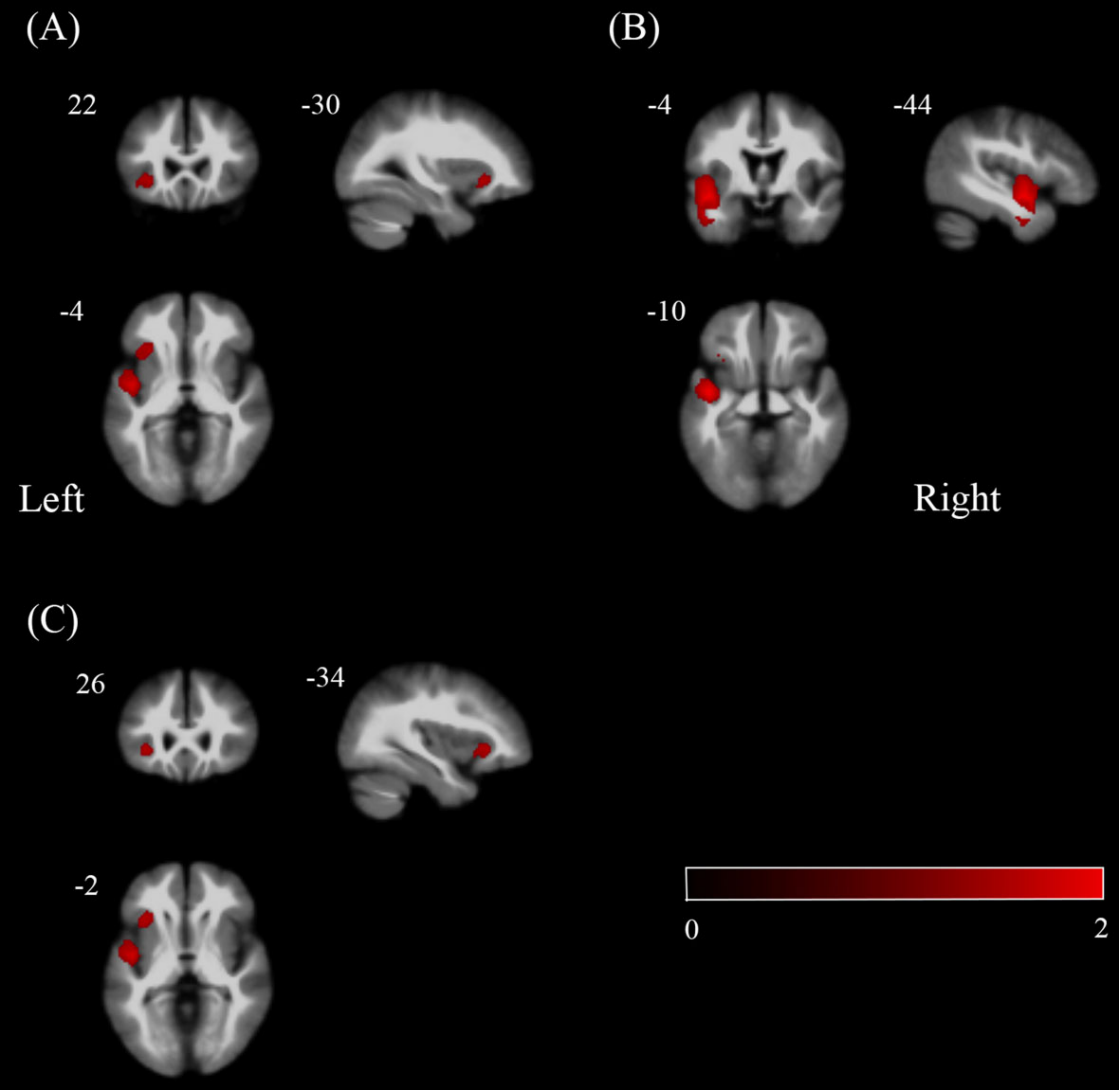
Areas with lower gray matter in Parkinson’s disease with mild cognitive impairment relative to Parkinson’s disease with normal cognitive function are displayed in red in (**A**) left insula, (**B**) left superior temporal gyrus, and (**C**) left inferior frontal gyrus. The color bar indicates the maximum and the minimum seed-based d mapping-Z values.

Voxel-based AES-SDM meta-analysis identified the temporal-insular-frontal lobe as the primary site of GM abnormalities associated with PD-MCI (Figure 2). Regions with significantly less GM were the left insula, left superior temporal gyrus, and left inferior frontal gyrus. Peak coordinates and cluster breakdowns are shown in Table 1. There were no regions showing increased GMV in PD-MCI compared to PD-NC patients.

**Table 1:** Clusters of gray matter volume reductions in patients with Parkinson’s disease with mild cognitive impairment compared with Parkinson’s disease with normal cognitive function.

Regions	No. of voxels	Maximum MNI coordinates	SDM-Z value	P value	Jackknife sensitivity analysis	P (Egger’s test)
Left insula	100	–30, 22, –4	–1.723	0.000099063	11 out of 12	0.250
Left superior temporal gyrus	914	–44, –4, –10	–2.176	0.000000536	10 out of 12	0.325
Left inferior frontal gyrus	147	–34, 26, –2	–1.726	0.000096500	6 out of 12	0.250

MNI: Montreal Neurological Institute; SDM: seed-based d mapping.

### Jackknife sensitivity and heterogeneity analyses

Systematic whole-brain jackknife sensitivity analysis of the PD-MCI findings revealed a significant reduction in GM in the left insula in 11 of 12 datasets. GM abnormalities in the left superior temporal gyrus were highly replicable and remained significant in 10 of 12 datasets (Table 1). None of the effect size differences in peak coordinates showed significant heterogeneity.

### Meta-regression

Meta-regression analyses revealed that age was negatively associated with GMV in the left rolandic operculum and that longer disease duration was correlated with greater GM atrophy in the right inferior temporal gyrus, left temporal pole, superior temporal gyrus, and left middle temporal gyrus. Additionally, higher UPDRS-III score and Hoehn and Yahr stage were correlated with greater GM atrophy in the left inferior temporal gyrus, right inferior temporal gyrus, left inferior frontal gyrus, and right middle frontal gyrus. There was no significant association between GMV reduction and education level or MMSE scores in PD-MCI patients. Detailed information is summarized in Table 2.

**Table 2:** Meta-regression analyses of demographic and clinical variables in Parkinson’s disease with mild cognitive impairment patients.

Variables/Anatomical regions	Peak MNI coordinates (x, y, z)	Number of voxels	SDM-Z value	P value
Age				
Left rolandic operculum	–50, 0, 4	1053	–2.401	0.000030994
PD duration				
Right inferior temporal gyrus	56, –20, –26	80	–1.415	0.000141382
Left temporal pole, superior temporal gyrus	–30, 20, –30	45	–1.518	0.000053644
Left middle temporal gyrus	–62, –24, 2	15	–1.361	0.000374138
UPDRS-III score				
Left inferior temporal gyrus	–46, –8, –40	106	–1.534	0.000009835
Right inferior temporal gyrus	46, –6, –40	39	–1.507	0.000207961
Left inferior frontal gyrus	–40, 34, 16	30	–1.513	0.000163078
Hoehn and Yahr stage				
Left inferior temporal gyrus	–46, –10, –36	88	–1.386	0.000248730
Right inferior temporal gyrus	48, –10, –38	85	–1.507	0.000128508
Left inferior frontal gyrus	–40, 36, 14	22	–1.318	0.000505269
Right middle frontal gyrus	38, 44, 12	17	–1.317	0.000588357
Education level	−	−	−	−
MMSE scores	−	−	−	−

MNI: Montreal Neurological Institute; SDM: seed-based d mapping; PD: Parkinson’s disease; UPDRS-III: Unified Parkinson’s Disease Rating Scale: Part III; MMSE: mini-mental state examination.

## Discussion

Our modified and comprehensive meta-analysis of 12 case-control VBM studies by AES-SDM showed that PD-MCI patients had less GM in the left superior temporal gyrus, left inferior frontal gyrus, and left insula. Moreover, the meta-regression analysis revealed that age, disease duration, UPDRS-III score, and Hoehn and Yahr stage were partly correlated with GM abnormalities in this group.

One of the most important findings of this study was that PD-MCI patients consistently showed significant reductions in GM in the left superior temporal gyrus, left inferior frontal gyrus, and left insula compared to PD-NC patients. The results of the meta-regression analysis indicated that decreased GM in these regions in PD-MCI patients was associated with older age, longer disease duration, and higher UPDRS-III score and Hoehn and Yahr stage. Imaging studies of cognitive impairment have shown GM reduction in fronto-temporal and posterior cortical areas (8,13,28,29–31). Importantly, GM abnormalities in the left temporal area were associated with impaired executive function, attention, and memory; those in the left frontal area were associated with impaired attention; and those in the left insular area were associated with impaired executive function, attention, and language abilities. This seems to suggest a possible relationship between cognitive decline in PD and structural decline in these brain regions, which is supported by the neuroanatomical abnormalities in the left superior temporal gyrus, left inferior frontal gyrus, and left insula identified in our meta-analysis. There is also some evidence that the frontal-limbic-temporal region (mainly the left temporal gyrus, left frontal gyrus, and left insula) is a potential therapeutic target in PD-MCI, although additional studies are needed to determine whether treatment reverses these structural changes and whether this morphometric pattern can serve as a prognostic biomarker.

The insula and temporal gyrus constitute the ventral stream of fronto-parietal pathways that play an important role in working memory and executive function (32). This stream is activated during attention shifting, interference resolution, and strategic organization (33); thus, atrophy of the insula and temporal gyrus can negatively impact frontal lobe-based cognitive performance. Our finding of a significant association between insula-temporal gyrus atrophy and deficits in executive function and attention in PD-MCI patients suggests early disruption of the ventral fronto-parietal pathway as a likely mechanism for the cognitive impairment observed in PD. Our results were consistent with those reported in a previous meta-analysis showing a decrease in signal intensity in the left temporal lobe extending to the left insula and left frontal lobe in patients with MCI compared with those without cognitive deficits (10). However, our meta-analysis included more studies than previous ones, which increased the statistical power. In addition, resting-state functional MRI revealed a reduced connectivity specifically in the default mode network (left precuneus, right median cingulate gyrus, left superior frontal gyrus, and right precentral gyrus) in PD-MCI patients (34). Interestingly, another recent meta-analysis found differences in several brain regions (left insula, bilateral dorsolateral prefrontal cortex, left angular gyrus, midcingulate cortex, and right supramarginal gyrus) between PD-MCI and PD-NC patients (35), which was not entirely consistent with our findings. This could be due to the fact that the earlier meta-analysis was biased as it pooled multiple studies that employed both VBM and cortical thickness measures, which may have reduced the sensitivity of detecting structural changes in subcortical regions such as the basal ganglia (35).

Interestingly, we did not consistently observe GM abnormalities in the hippocampus, which is a hallmark of Alzheimer’s disease (36) even though over half of PD patients exhibit Alzheimer’s disease-related neuropathologic changes in the hippocampus that are detectable by autopsy (37). Hippocampal GMV reduction has been linked to hippocampal neuron atrophy in the MCI stage of PD (38). This discrepancy may be attributed to the limited number of studies that were included in our meta-analysis, sample features, or methodological factors. It has been reported that neurodegeneration in PD patients begins in the head and spreads to the tail of the hippocampus (39). However, the results of our study indicated that hippocampal atrophy was not closely associated with the initial stages of cognitive dysfunction in PD.

Our study had several limitations. First, the sample sizes of included studies were relatively small, and therefore the results must be interpreted with caution. On the other hand, a large number of studies were included and our findings can be considered robust because nearly all of the studies contributed to the results. Second, the heterogeneity of VBM study methodology including different preprocessing protocols and statistical thresholding methods may have influenced the results. Third, the results of the meta-regression analysis also found longer disease duration was correlated with greater GM atrophy in the left superior temporal gyrus. This seems to suggest the degenerative course of the disease in PD also may have occurred along grey matter loss in the left superior temporal gyrus, which may have led to less accurate results. However, a recent meta-analysis found that GM reductions were detected in the right temporal lobe, right rolandic operculum, right amygdala, right angular gyrus, right middle occipital gyrus, right fusiform gyrus, left superior frontal gyrus, bilateral anterior cingulate/paracingulate gyri, bilateral insula, and bilateral striatum in the PD patients compared with the healthy controls. Regions with significantly less GM were the left insula, left superior temporal gyrus, and left inferior frontal gyrus. No significant regional GM atrophy in left superior temporal gyrus was detected in the PD patients (40).

In conclusion, the present voxel-wise meta-analysis revealed that GM was reduced in the left insula and left superior temporal gyrus of PD-MCI patients compared with PD-NC patients. Furthermore, we found that age, disease duration, UPDRS-III scores, and Hoehn and Yahr stage were partly correlated with GM abnormalities in PD-MCI patients. These findings may provide a basis for investigations on the mechanisms underlying changes in the visual cortex that are associated with cognitive decline in PD. Future longitudinal and multicenter MRI studies should investigate whether this brain morphometric pattern can serve as a useful target and a prognostic marker for PD-MCI diagnosing and treatment.

## Supplementary Material

Click here to view [pdf].
